# Effect of H_2_O_2_-V_C_ degradation on structural characteristics and immunomodulatory activity of larch arabinogalactan

**DOI:** 10.3389/fbioe.2024.1461343

**Published:** 2024-08-07

**Authors:** Huimin Qi, Shuo Tang, Bin Bian, Chenhuan Lai, Yanan Chen, Zhe Ling, Qiang Yong

**Affiliations:** ^1^ Jiangsu Co-Innovation Center of Efficient Processing and Utilisation of Forest Resources, College of Chemical Engineering, Nanjing Forestry University, Nanjing, China; ^2^ Nanjing Institute of Comprehensive Utilization of Wild Plants, Nanjing, China

**Keywords:** arabinogalactan, degradation, H_2_O_2_-VC, structural characteristics, immunomodulatory activity

## Abstract

The arabinogalactan in the representative softwood biomass of larch was degraded using an environmentally friendly hydrogen peroxide and vitamin C (H_2_O_2_-V_C_) system to improve its immunomodulatory activity. Through the H_2_O_2_-V_C_ degradation mechanism, hydroxyl radicals are generated, which then target the hydrogen atoms within polysaccharides, resulting in the breaking of glycosidic bonds. Given the impact of oxidative degradation on polysaccharides, we identified three specific arabinogalactan degradation products distinguished by their arabinosyl side chain compositions. The primary structures of the degradation products were investigated using Fourier-transform infrared spectroscopy and nuclear magnetic resonance spectroscopy. Congo red staining showed that the degradation products were absent in the triple-helix structure. The results of the *in vitro* immunological experiments indicated that an appropriate reduction in the molar ratio of arabinose to galactose enhanced the immunostimulatory effects on RAW 264.7 cells. In addition, the immunostimulatory pathway mediated by arabinogalactan was explored by toll-like receptor 4 (TLR4) inhibitor (TAK-242) These findings provide novel insights into the understanding of the relationship between the structure of arabinogalactan and its biological activity.

## 1 Introduction

The larch, a deciduous coniferous lignocellulosic biomass of the genus *Pinus* in the family Pinaceae, is native to China and is primarily found in Inner Mongolia and the Provinces of Jilin, Liaoning, and Heilongjiang. The tree provides wood for a wide range of items, including utility poles, bridges, and mining pillars, as well as lumber used in shipbuilding, vehicle manufacture, and general construction and pulp for papermaking (Bai et al., 2021). The felling and processing of larch inevitably produce wood residues, which can be utilized to produce valuable products. Interestingly, larch has been found to be abundant in water-soluble arabinogalactan (AG). The structure of AG in larch has been investigated in a previous study (Tang et al., 2018). AG is a water-soluble polysaccharide characterized by a high degree of branching. Specifically, AG consists of galactose and arabinose, with a main chain constituted by β-1,3-linked galactose residues, with branches at the O-6 position of the main chain into mono- or oligosaccharide side chains composed of Gal and Ara.

AG from larch has been verified to have anti-tumor and immune activities and promotes the growth of intestinal microbes (Currier et al., 2003; Rakhmanberdyeva et al., 2019). Therefore, the extraction of AG from larch is not only an important method of waste utilization, but also provides polysaccharides as important material in the food and pharmaceutical industries (Xu et al., 2018). The results of some studies suggest that the degraded components can effectively promote the secretion of pro-inflammatory cytokines (Cicinskas et al., 2019). Notably, the side-chain degradation and chain conformation elongation of AG may exhibit enhanced immunomodulatory activity (Cheng et al., 2021). Furthermore, it has been demonstrated that the degradation of *Lycium barbarum* polysaccharides results in a notable enhancement of their anti-inflammatory activity. This is achieved by increasing the levels of nitric oxide (NO), phagocytosis, and acid phosphatase in RAW 264.7 cells (Gong et al., 2018). The enhanced immunomodulatory activity of degraded polysaccharides may potentially be attributed to their capacity to induce Toll-like receptor 4 (TLR4) receptors on macrophage surfaces (Zhang X. R. et al., 2014). These results indicated that optimized degradation of polysaccharides could result in enhanced beneficial activity (Liang et al., 2023; Wang et al., 2024). Therefore, we hypothesized that partial degradation of AG could potentially improve its biological activity.

In recent years, multiple physical, biological, and chemical methods have been proposed for polysaccharide degradation. Physical degradation, via ultrasound or radiation, is a time-saving and easy technique (Hu et al., 2023). However, the cost of physical degradation is high and the product yield is low. Biodegradation, primarily enzymatic degradation, is a rapid, gentle, and nontoxic method for degrading polysaccharides (Drouillard et al., 2022); nevertheless, the high cost and instability of enzymes represent significant limitations to their broader application. Moreover, there are few reports on AG-degrading enzymes, which limits the study of the enzymatic degradation of AG (Cheng et al., 2023; Fan et al., 2023). Chemical degradation includes oxidative degradation (Ma et al., 2021) and acidic or alkaline hydrolysis (Zheng et al., 2021). Acid or alkali hydrolysis makes it difficult to purify and pollutes the environment, whereas oxidative degradation is an ideal method for polysaccharide degradation because of its simplicity, low requirements for degradation equipment, non-toxicity, and lack of by-products.

H_2_O_2_ oxidation has been widely used in polysaccharide degradation in recent years. The process—which involves hydroxyl radicals, generated by H_2_O_2_ decomposition, that oxidatively break polysaccharide glycosidic bonds—is regarded as a green and controllable method (Sun et al., 2015). The spontaneous generation of hydroxyl radicals by H_2_O_2_ slowly degrades polysaccharides. In the presence of both H_2_O_2_ and V_C_, ascorbic acid generates hydroxyl radicals via the Fenton reaction, which attack polysaccharide molecules and initiate a series of reactions, including depolymerization (Wang et al., 2023). Hydroxyl radicals can also be produced by oxidizing ascorbic acid with hydrogen peroxide, resulting in the production of 2,3-diketogu-lonic acid (Curcio et al., 2009). These hydroxyl radicals are highly reactive and react with the hydrogen atoms of the polysaccharide (hydrogen extraction reaction), leading to glycosidic bond cleavage (Chen et al., 2021). In contrast, the addition of a low concentration of V_C_ significantly increases the number of free radicals, and the rate of polysaccharide degradation is significantly faster than that of the single H_2_O_2_ system (Zou et al., 2020).

In the present study, we selected the H_2_O_2_-V_C_ method used to degrade AG, after which the structural characteristics of the degradation products were analyzed. In addition, RAW 264.7 cells were utilized to evaluate the immunoreactivity of AG degradation products. We report the successful development of an alternative extraction method for AG oligosaccharides and key insights into the effect of arabinose removal on the immunological activity of AG polysaccharides.

## 2 Materials and methods

### 2.1 Materials and reagents

Larch material was sourced from the Greater Khingan Mountains in Northeast China and was provided by the Beijing Yucheng Wood Processing Factory. Mouse macrophage (RAW 264.7) cells were obtained from the Shanghai Institute of Biological Sciences, Chinese Academy of Sciences (SIBS). Dulbecco’s modified Eagle medium (DMEM; CAS: C11995), fetal bovine serum (FBS; CAS: 11011-8611), and phosphate buffered saline were purchased from Hyclone, United States. Lipopolysaccharide (LPS; CAS: L2880) and dimethyl sulfoxide were purchased from Sigma-Aldrich. The Cell Counting Kit (CCK-8; CAS: BS350C) and nitric oxide (NO; CAS: S0021S) assay kits were procured from Beyotime. The ELISA kits for mouse interleukin 6 (IL-6; CAS: 70-EK206/3-96) and tumour necrosis factor-α (TNF-α; CAS: 70-EK282/4-96) were purchased from Multi Sciences Biological Company. Other reagents, such as ascorbic acid, 30% hydrogen peroxide, anhydrous ethanol, Congo red, and potassium dihydrogen phosphate, were procured from Sinopharm Chemical Reagent Company Limited and were of analytical grade.

### 2.2 Preparation and purification of AGR

Larch wood was crushed using a pulverizer, and the wood chips were passed through a 20–80-mesh sieve. The aforementioned material was then combined with distilled water in a 1:10 ratio (g/g) and subjected to continuous stirring at a temperature of 60°C for a period of 2 h. The mixture of solid and liquid was then filtered through 300-mesh gauze to separate the solid and liquid portions, the liquid portion being the crude polysaccharide solution. The solution was then subjected to filtration by means of pumping and centrifugation (8,000 rpm, 5 min) in order to obtain the supernatant. This was then concentrated by a factor of 10 using a rotary evaporator. The concentrated sugar solution was eluted through a 15 cm × 1 cm glass column containing 25 g of XAD-16N resin, and the eluate was collected twice. Three times the volume of 95% ethanol was added to the concentrated solution, which was then left overnight to precipitate at 4°C. The precipitate was obtained via centrifugation (8,000 rpm, 5 min) and freeze-drying, which yielded the polysaccharide powder. The extracted AG was designated as AGR.

### 2.3 Preparation of AGR degradation products

The AGR was subjected to degradation via the H_2_O_2_-V_C_ system, in accordance with the methodology delineated in a preceding study (Yao et al., 2013). In summary, 5 mg/mL AG solution, 0.05–0.8 M H_2_O_2_, and 10–80 mM V_C_ were combined and allowed to react at 60°C, 100°C, and 140°C for 0.5–4 h in a final volume of 40 mL. After the degradation reactions were complete, the pH of the reaction system was adjusted to neutral. The resulting solution was dialyzed in deionized water (200 Da) for a period of 48 h, concentrated by distillation under reduced pressure, and the AGR degradation product was then lyophilised. The degradation rate of the three different degradation products (DAG-60, DAG-100, and DAG-140) was calculated as the mass difference between the pre- and post-degradation mass/pre-degradation mass.

$$\text{Degradation rate}\left(\%\right)=\frac{\text{pre}‐\text{degradation}\;\text{mass}\;‐\;\text{post}‐\text{degradation}\;\text{mass}}{\text{pre}‐\text{degradation}\;\text{mass}}$$

### 2.4 Characterization of AGR and diacylglycerols (DAGs)

#### 2.4.1 Quantitative analysis of AGR and DAGs

The degraded and undegraded AGR were determined via one-step sulfuric acid hydrolysis (Gao et al., 2014). Specifically, AGR samples (0.3 ± 0.01 g, dry weight) were directly hydrolyzed with 4% (w/w) sulfuric acid for 1 h at 121°C, followed by neutralisation with NaOH and centrifugation (8000 rpm, 5 min). The resulting supernatant was filtered through a filter and injected into the Dionex ICS-5000 system at 30°C. (Thermo Fisher Scientific, Waltham, MA, United States). The samples were calibrated using standard external sugar solutions of L-arabinose and D-galactose.

#### 2.4.2 Measurement of protein and ash content

The protein content of the AGR was quantified using a Bio-Rad protein assay, with bovine serum albumin (BSA) serving as the standard. The absorbance was quantified at 595 nm using a UV-visible spectrophotometer (UV-1800, Shimadzu, Tokyo, Japan) and a 1 cm wide quartz cuvette. The ash content of the AGR was analyzed according to AACC method 08-01 (Consumi et al., 2022).

#### 2.4.3 Molecular weight analysis

The molecular weight distributions of the three degradation products and unmodified AGR were determined via gel permeation chromatography (GPC, Agilent Technologies, Santa Clara, California, United States) on a column equipped with a refractive index detector and three tandem columns. The mobile phase (ultrapure water) was used at a flow rate of 0.6 mL/min. Samples were then diluted to approximately 1.0 g/L and then filtered through a filter before injection. Dextran standards with a mean molecular weight of 1.80 × 10^2^ Da–6.7 × 10^5^ Da were employed to calibrate the column at 65°C during the separation process. The weight-average molecular weight (Mw), number-average molecular weight (Mn), and polydispersity index (PDI = Mw/Mn) were calculated using the Agilent GPC analysis software for Agilent ChemStation.

#### 2.4.4 Fourier-transform infrared spectroscopy (FTIR) analysis

The functional groups present in the three degradation products and the undegraded AGR were analyzed via FTIR using a spectrometer (VERTEX 80 V, Bruker, Ettlingen, Germany) and the KBr-disk method. The spectra were recorded within the range of 4,000–400 cm^−1^.

#### 2.4.5 Nuclear magnetic resonance (NMR) analysis

Each polysaccharide (50 mg) was dissolved in deuterium oxide (D_2_O, 0.6 mL) at room temperature. The standard employed was 4,4-dimethyl-4-silylpentane-1-sulfonic acid. ^13^C NMR spectra were obtained using a Bruker AVANCE 600 MHz spectrometer (Bruker, Bremen, Germany) with a standard Bruker pulse sequence at a temperature of 25°C.

#### 2.4.6 Congo red assay

The solution conformation of polysaccharides was determined using special binding of Congo red to triple helix polysaccharides. A 2-mL volume of polysaccharide solution (2 mg/mL) was combined with 2 mL of Congo red solution (0.2 mM) and 1 mL of NaOH at varying concentrations, with the final NaOH concentration ranging from 0 to 1 M. The solution was kept in the dark for 10 min, after which the maximum absorption wavelength (λmax) was measured in the range of 400–600 nm using a UV-1800.

### 2.5 Assessment of immunomodulatory activity

#### 2.5.1 Cell culture

RAW 264.7 cells were cultured in DMEM supplemented with 1% (v/v) double antibody (a mixture of penicillin and streptomycin) and 10% (v/v) FBS. The cells were cultured in a humidified atmosphere at 37°C, which contained 5% CO_2_.

#### 2.5.2 Cell viability assay

RAW 264.7 cells (1 × 10^5^ cells/mL) were inoculated in 96-well plates and treated with different concentrations (25, 50, 100, 200, 400, and 800 μg/mL) of degraded polysaccharides for 24 h to detect the effect of DAGs and undegraded AGR on RAW 264.7 cell viability. Cell viability was determined using the CCK-8 assay according to the manufacturer’s protocol. LPS (1 μg/mL) and complete medium were used as positive and control groups, respectively. Each treatment consisted of three replicates.

#### 2.5.3 NO production

The Griess reaction was employed to determine whether RAW 264.7 cells exhibited any production in response to DAGs and AGR. In brief, RAW 264.7 cells (1 × 10^5^ cells/mL) were inoculated in 96-well plates, incubated for 24 h, and then treated with different concentrations of AGR and DAGs. LPS (1 μg/mL) and complete medium were employed as the positive and control groups, respectively. Following a 24 h incubation period, the NO concentration in the conditioned medium was quantified using an NO assay kit. Each treatment was conducted in triplicate.

#### 2.5.4 Secretion of TNF-α and IL-6

RAW 264.7 cells (1 × 10^5^ cells/mL) were inoculated into 6-well plates and incubated with different concentrations of AGR, DAG, or LPS (1 μg/mL) for an additional 24 h. Following centrifugation, the supernatant was collected, and the concentrations of TNF-α and IL-6 were measured using ELISA kits according to the manufacturer’s protocol. Each sample was detected three times in one experiment.

### 2.6 Effect of TAK242 on the immunoreactivity of AGR and DAGs in RAW 264.7 cells

#### 2.6.1 Effect of TAK-242 on RAW 264.7 cell viability

Briefly, 100 μL of RAW 264.7 cells in logarithmic growth phase (1 × 10^5^ cells/mL) were inoculated into 96-well plates and cultured for 24 h. After removing the culture medium, 100 μL of fresh basal medium containing different concentrations of TAK-242 (0.5–64 μM) was added. The culture was incubated in basal medium without TAK-242 as a control. Cell viability was analyzed after 4 h of incubation using a CCK-8 kit to determine the toxicity of different concentrations of TAK-242. All experiments were performed in triplicates.

#### 2.6.2 Effect of TAK242 on the secretion of NO and cytokines by RAW 264.7 cells stimulated by AGR and DAGs

A 100-µL volume of logarithmic growth phase RAW 264.7 cells (1 × 10^5^ cells/mL) was deposited into 96-well plates and cultured for 24 h. After removing the medium, fresh complete medium with and without TAK-242 (2 μM) was added to the wells and cell culture continued for 4 h. The cells were then incubated for 24 h with DAGs (800 μg/mL) and positive control LPS (1 μg/mL). The levels of NO, TNF-α, and IL-6 in the cell supernatant were determined using Griess assay kit and ELISA kit.

### 2.7 Statistical analysis

The results were expressed as mean ± standard deviation. Experimental data were analyzed via one-way ANOVA using SPSS 21.0. Post-hoc tests were performed using Tukey’s honest significant difference test. *P* < 0.05 was considered statistically significant.

## 3 Results and discussion

### 3.1 Characterization of AGR

As shown in Table 1, the AGR extracted from larch wood was of high purity (93.79%), and the ratio of arabino-to-galactose (A/G) was 1:9.06. Owing to the differences between larch species from different geographical regions, the proportion of monosaccharide residues of AG in larches from different origins varies slightly. Despite the high purity of the extracted AGR, there were still minor impurities present, which may include phenolic compounds, as well as traces of glucuronic acid and rhamnose (Tang et al., 2018; Guo et al., 2021; Machado et al., 2024). It has been documated that polysaccharides of high purity generally exhibit enhanced immunological activity (Chen and Huang, 2018). Therefore, the extracted AGR were further isolated and purified.

**TABLE 1:** Contents of arabinogalactan (AGR) extracted from larch wood.

Sample	Arabinogalactan (%)			Ara:Gal	Protein (%)	Ash (%)	Impurities (%)
	Ara^a^	Gal^b^	All^c^				
AGR	10.97	82.82	93.79	1:9.06	0.05	1.10	5.06

^a^ arabinose.

^b^ galactose.

^c^ sum of arabinose and galactose.

### 3.2 Preparation of AGR degradation products

The biological activity of polysaccharides extracted from plants are significantly limited by their complex conformations. Research has demonstrated that partially degraded polysaccharides exhibit enhanced bioactivity compared with their undegraded counterparts. Hence, appropriate degradation of polysaccharides is essential for enhancing their bioactivity. Among the various degradation methods, the H_2_O_2_-Vc system was selected for this study because of its environmental friendliness and mild reaction conditions.

To obtain partially degraded AGR,–H_2_O_2_-V_C_, oxidation experiments were performed under different reaction conditions: concentration of H_2_O_2_ and V_C_, reaction time, and temperature.

**FIGURE 1:**
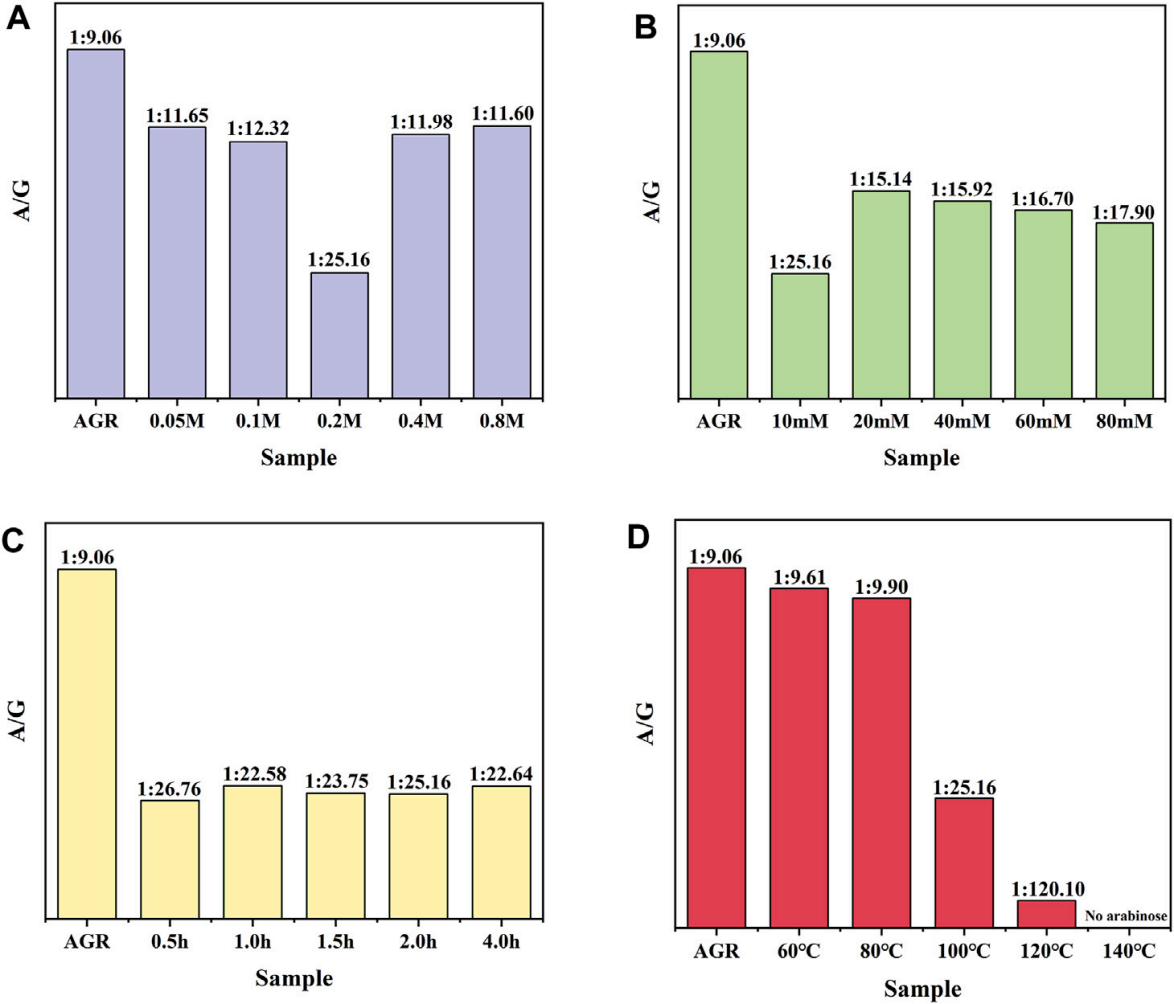
Effect of H_2_O_2_-VC degradation conditions on the molar ratio of arabinose to galactose (A/G): **(A)** H_2_O_2_ concentration, **(B)** VC concentration, **(C)** time, and **(D)** temperature.

First, a one-factor experiment was performed with a series of H_2_O_2_ concentrations (0.05, 0.1, 0.2, 0.4, and 0.8 M) and a fixed V_C_ concentration (10 mM) at 100°C for 2 h. As the concentration of H_2_O_2_ increased, degradation of the samples initially increased and then decreased. This reduction can also be attributed to excessive hydrolysis of H_2_O_2_ in the presence of excess H_2_O_2_. The degradation rate of the samples reached a maximum value of 38.83% under the condition of 0.2 M H_2_O_2_ treatment (Supplementary Figure S1). Changes in the Mw and A/G values exhibited a similar pattern. Specifically, the sample treated with 0.2 M H_2_O_2_ had the lowest A/G value, at 1:25.16 (Figure 1) and the lowest Mw, 15 kDa (Table 2). Based on previous findings, A/G and Mw may serve as important indicators to evaluate the AGR degradation products. Treatment with H_2_O_2_ (5–150 mmol/L) and UV radiation enhanced the degradation of *Sargassum* polysaccharides in a concentration-dependent manner; however, the increase in degradation efficiency may become negligible at H_2_O_2_ concentrations above 75 mmol/L (Chen et al., 2020). This result was attributed to the fact that excess H_2_O_2_ can lead to the reduction of hydroxyl radicals in such systems, thereby reducing the degradation efficiency of the polysaccharides. Degradation reduces the molecular weight of polysaccharides and affects their physicochemical properties and spatial conformation, thereby altering their structure and improving their biological activity (Ai et al., 2012). Combining this evidence, the sample degraded by 0.2 M H_2_O_2_ with lower A/G and Mw may exhibit better biological activity. Therefore, we chose the concentration of H_2_O_2_ as 0.2 M.

**TABLE 2:** Relative molecular weight parameters of degradation products at different H_2_O_2_ concentrations.

Sample	Mn (kDa)	Mw (kDa)	PDI
AGR	17.6	18.1	1.03
0.05 M	15.4	15.6	1.01
0.1 M	15.4	15.5	1.00
0.2 M	14.9	15.0	1.00
0.4 M	15.0	15.2	1.01
0.8 M	15.2	15.3	1.00

AGR degradation experiments were continued with a fixed H_2_O_2_ concentration (0.2 M) and a series of V_C_ concentrations (10, 20, 40, 60, and 80 mM) at 100°C for 2 h. When the V_C_ treatment concentration of V_C_ was increased from 5 to 10 mM, the degradation rate of AGR increased sharply from 22.85% to 49.37% and did not change significantly when the treatment concentration of V_C_ continued to increase (Supplementary Figure S2). This indicates that the addition of 10 mM V_C_ was sufficient to degrade AGR. Another study confirmed that a lower concentration of V_C_ was more effective in degrading polysaccharides through hydroxyl radicals (Ofoedu et al., 2021). Notably, the AGR degradation products treated with 10 mM V_C_ had a lower A/G ratio and molecular weight of 1:25.16 (Figure 1) and 15 kDa (Table 3), respectively, than the AGR degradation products treated with higher concentrations of V_C_. Interestingly, increasing the concentration of ascorbic acid from 1 to 10 mM also improved the degradation efficiency of pectin polysaccharides; however, concentrations higher than 10 mM of ascorbic acid failed to improve the degradation efficiency. This was mainly due to the depletion of hydroxyl radicals generated by excess ascorbic acid molecules (Li et al., 2019). Overall, 10 mmol of V_C_ concentration was selected.

**TABLE 3:** Relative molecular weight parameters of degradation products by V_C_ concentration gradient.

Sample	Mn (kDa)	Mw (kDa)	PDI
AGR	17.2	17.7	1.03
10 mM	14.8	15.4	1.03
20 mM	16.4	16.7	1.02
40 mM	15.9	16.4	1.03
60 mM	16.3	16.8	1.01
80 mM	16.4	16.6	1.01

To further determine the appropriate time for H_2_O_2_-Vc treatment, AGR degradation experiments were continued at 100°C for 0.5, 1, 1.5, 2, and 4 h at fixed H_2_O_2_ concentration (0.2 M) and V_C_ concentration (10 mM). The degradation rate of AGR gradually increased with increasing treatment time from 0.5 to 2 h (Supplementary Figure S3), whereas the Mw of the AGR degradation products did not significantly change (Table 4). However, when the treatment time was extended to 4 h, the changes in the Mw, monosaccharide content, and degradation rate of the AGR degradation products were minimal compared with the 2 h treatment time. Previous research has shown that the reaction time is only effective for a certain time period and that extending the reaction time beyond that time period has little or no effect on the degradation of polysaccharides, which may be due to the depletion of free radicals during that time period (Liang et al., 2017). The A/G of the AGR degradation products obtained by treatment with H_2_O_2_-Vc was significantly reduced, regardless of the treatment duration. In particular, the A/G of the product degraded for 2 h reached the lowest value of 1:25.16 (Figure 1). Therefore, 2 h was selected as the optimal time for H_2_O_2_-Vc treatment.

**TABLE 4:** Relative molecular weight of degradation products at different reaction times.

Sample	Mn (kDa)	Mw (kDa)	PDI
AGR	18.2	18.7	1.03
0.5 h	15.2	15.5	1.02
1.0 h	14.4	14.7	1.02
1.5 h	13.9	14.4	1.03
2.0 h	14.2	14.3	1.01
4.0 h	14.1	14.4	1.02

Lastly, AGR degradation experiments were performed at temperatures (60°C, 80°C, 100°C, 120°C, and 140°C) under fixed H_2_O_2_ concentration (0.2 M) and V_C_ concentration (10 mM) for 2 h to determine the appropriate temperatures for H_2_O_2_-Vc treatment. Reaction temperature is a key factor in polysaccharide degradation because it determines the rate of the reaction. High temperatures (but not excessive temperatures) usually have a positive effect on polysaccharide degradation because more energy is provided to activate the reaction. With increasing treatment temperature, the AGR degradation rate gradually increased (Supplementary Figure S4), whereas the A/G (Figure 1) and Mw (Table 5) gradually decreased. Notably, the side-chain arabinosyl groups of AGR were almost completely removed at a treatment temperature of 140°C. Therefore, different structural features were observed among the samples treated at different temperatures using H_2_O_2_-Vc. In particular, the samples obtained under 60°C, 100°C, and 140°C (labeled DAG-60, DAG-100, and DAG-140) exhibited significant variability in A/G, as well as differences in Mw. We further utilized the RAW 264.7 cellular immunity model to assess the *in vitro* immunomodulatory activity of DAG-60, DAG-100, and DAG-140.

**TABLE 5:** Relative molecular weight of degradation products at different reaction temperatures.

Sample	Mn (kDa)	Mw (kDa)	PDI
AGR	18.2	18.7	1.03
60°C	16.3	16.7	1.02
80°C	15.5	16.0	1.03
100°C	14.7	15.0	1.02
120°C	12.8	13.2	1.03
140°C	12.6	12.9	1.02

### 3.3 FT-IR and NMR analysis

**FIGURE 2:**
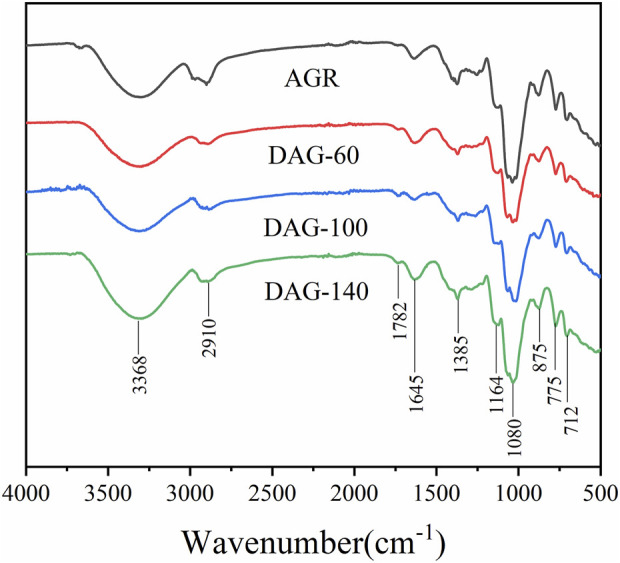
Fourier transform - infrared spectroscopy (FT-IR) of AGR and degraded products.

To further investigate the chemical structure of the AGR degradation products, FTIR studies were performed on DAG-60 (Ara:Gal ratio of 1:9.61), DAG-100 (Ara:Gal ratio of 1:25.16), and DAG-140 (arabinose-free). An additional NMR study was performed using AGR and DAG-140. As shown in Figure 2, typical polysaccharide absorption peaks at 3,368, 2,910, 1,645, and 1,080 cm^−1^ were observed in all the AG-degraded samples. After an in-depth analysis of the signal peaks, it can be concluded that the main structure of the polysaccharide remained intact, which is related to the relatively mild structure of AG in the H_2_O_2_-V_C_ degradation system (Zhang Z. S. et al., 2014). The presence of absorption peaks in the 1,200–1,000 cm^−1^ range suggests a pyranose unit (Yuan et al., 2020). The 875 cm^−1^ peak corresponds to the *β*-glycosidic bond. However, some minor variations were observed in the regions of 3,600–3,200 and 1,200–1,000 cm^−1^. The absorption peaks of the degraded polysaccharides were more intense than those of AGR around 3,368 cm^−1^, in contrast to DAG-100 at 1,782 cm^−1^, which was more pronounced than that of the other samples, and DAG-140 at 1,782 cm^−1^, which was significantly smaller than that of DAG-100. The absorption peak of DAG-140 at 1,782 cm^−1^ was significantly smaller than that of DAG-100, which may be related to the formation of O-H groups during the degradation process (Li et al., 2020). FTIR analysis revealed that polysaccharides degraded by H_2_O_2_-V_C_ retained their glycan structure.

**FIGURE 3:**
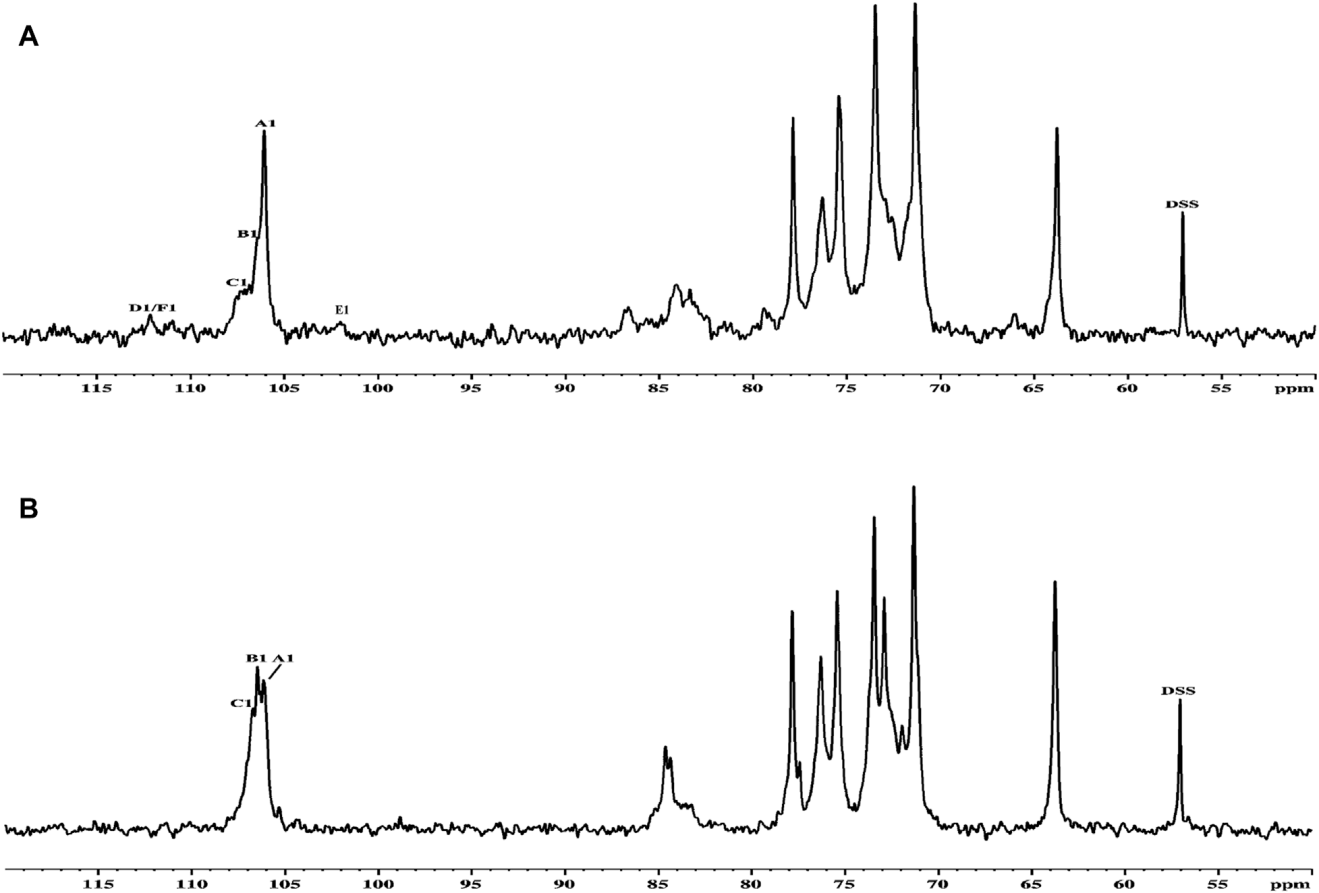
^13^C NMR spectra of AGR **(A)** and DAG-140 **(B)**.

Thus, the investigation was supported via NMR spectroscopy to determine the structural patterns of the products of AGR degradation. All ^13^C NMR signals of the sugar moieties were completely assigned, as shown in Table 6. Six typical signals were found at 107.05, 72.95, 84.67, 71.14, 77.68, and 63.82 ppm, suggesting that these signals belonged to the →3)-Galp-(1→ residues of C-1, C-2, C-3, C-4, C-5 and C-6 respectively (Chen et al., 2017; Trigui et al., 2018). Compared with the carbon spectrum of the original AGR (Figure 3A), there was a distinct peak at 84.67 ppm in the carbon spectrum of DAG-140 (Figure 3B). In addition, DAG-140 lost the signals at 101.98 and 112.09 ppm. This observation suggests that new sugar residues are generated during H_2_O_2_-V_C_ degradation and that terminal sugar residues are removed. This was due to the removal of the branch structure, which exposed new portions of the main polysaccharide chain of the AGR. Thus, NMR spectroscopy (^13^C NMR) data indicated that H_2_O_2_-V_C_ degradation removed some galactose residues in the side chain and all arabinose residues from the side chain.

**TABLE 6:** ^13^C NMR chemical shifts (ppm) in AGR and DAG-140.

Residues	C-1	C-2	C-3	C-4	C-5	C-6
(A) T-β-D-Gal*p*	106.06	73.54	75.66	71.99	78.06	63.87
(B) →6)-β-D-Gal*p*-(1→	106.10	73.60	74.54	71.56	77.74	72.02
(C) →3,6)-β-D-Gal*p*-(1→	106.84	73.07	84.15	71.56	77.70	72.96
(G) →3)-β-L-Gal*p*-(1→	107.05	72.95	84.67	71.14	77.68	63.82
(D) T-α-L-Ara*f*	112.09	83.54	79.52	86.34	64.01	
(E) T-β-L-Ara*p*	101.98	70.77	70.77	71.57	66.48	
(F) →3)-α-L-Ara*f*-(1→	111.10	82.89	86.84	86.59	64.01	

### 3.4 Congo red assay

Congo red experiments were conducted to further determine the triple-helix structure of the degraded products of AGR. As shown in Supplementary Figure S5, the maximum absorption wavelength of the Congo red-AGR complex gradually decreased as the concentration of NaOH gradually increased, approaching the maximum absorption wavelength of Congo red, indicating that the alkaline solution disrupted the hydrogen bonding of the polysaccharide. However, the presence of polysaccharide degradation products in the Congo red solution resulted in a significant shift in the maximum absorption wavelength from 497 to 502 nm and 504 nm, indicating the presence of polysaccharide-Congo red complexes. At the same NaOH concentration, all degraded AGR product complexes showed a red-shift compared to Congo red. However, there was no specific shift in the maximum absorption wavelength at different concentrations of NaOH. These results suggested that the H_2_O_2_-V_C_ degraded AGR did not show a triple helix conformation in solution. We speculated that the triple helix structure may be difficult to form in the polysaccharides with low molecular weight, but this view still needs further studies to verify.

### 3.5 Immunomodulatory activity of the degradation products

#### 3.5.1 Effect of AGR and DAGs on RAW 264.7 cell viability and NO production

**FIGURE 4:**
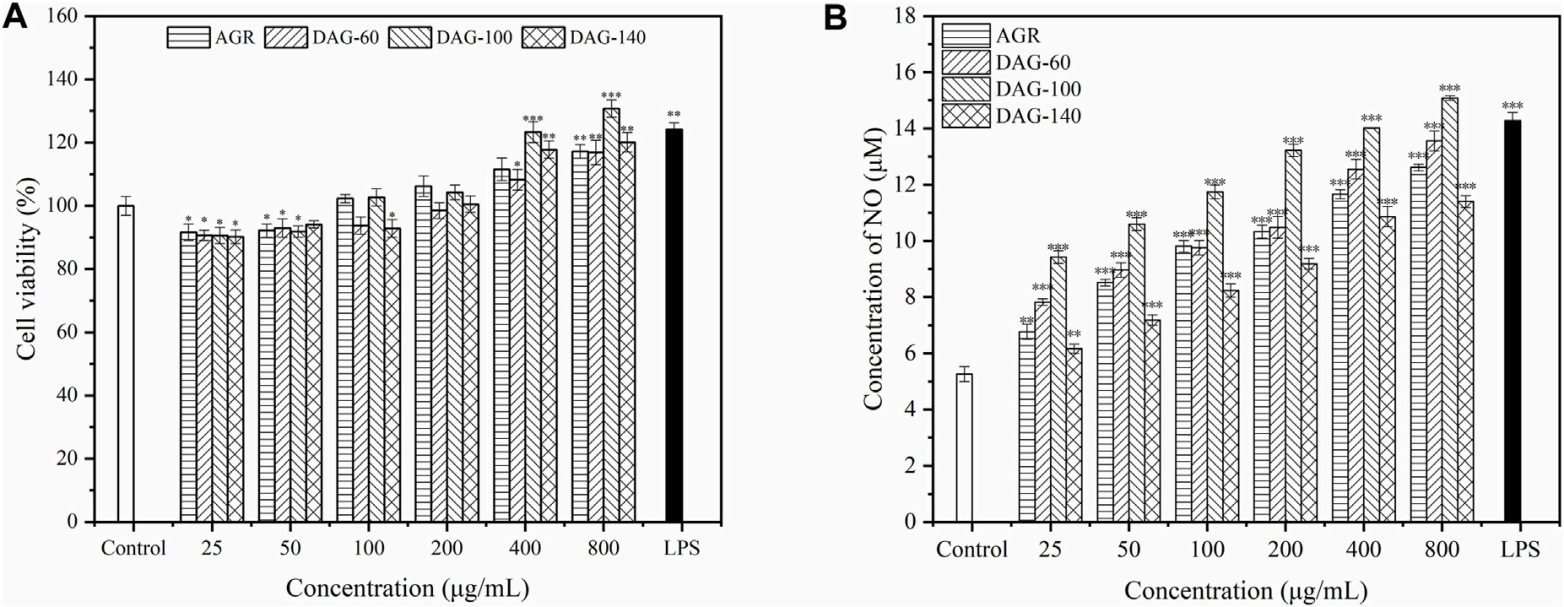
Effect of AGR and DAGs on RAW 264.7 cell survival **(A)** and NO production **(B)** *P < 0.05, **P < 0.01, ***P < 0.001 compared to the control.

Macrophages, an important component of the immune system, play an important role in the clearance of pathogens and maintenance of tissue homeostasis, and are the most common models for investigating the immunomodulatory activity of polysaccharides (Wang et al., 2014). In the present study, RAW 264.7 cells were subjected to different concentrations of polysaccharide solutions ranging from 25 to 8,00 μg/mL to determine their effect on cell viability. The effect of the three degradation products on the proliferation of RAW 264.7, cultured with the AGR degradation product, is shown in Figure 4A. AGR and DAGs (a general term for DAG-100, DAG-60, and DAG-140) at concentrations of 25–100 μg/mL exerted significant cytotoxicity in RAW 264.7 cells (*P* < 0.05), except for DAG140 at 100 μg/mL. The toxic effect of AGR and DAGs on RAW 264.7 cells began to diminish when the treatment concentration was increased to 200 μg/mL. At treatment concentrations of 400 and 800 μg/mL, DAGs promoted the proliferation of RAW 264.7 cells in a concentration-dependent manner compared to the control group. DAG-60 exerted comparable proliferative impact on RAW 264.7 cells to that of AGR. This equivalence in biological activity was likely due to the analogous A/G observed between the two compounds, with AGR having an A/G value of 1:9.06 and DAG-60 a similar value, 1:9.61. Lo et al. showed that polysaccharides with similar glycosyl ratios exhibited similar cell proliferative abilities (Lo et al., 2011). DAG-100 exhibited the best cell proliferation ability with a lower A/G. However, DAG-140, without side chains, showed a weaker proliferation effect on RAW 264.7 cells than DAG-100, indicating that the side chain of AGR plays a non-negligible role in the regulation of cell proliferation process.

NO is an important factor stimulated and secreted by immune cells. It regulates macrophage function, growth, and differentiation, thereby participating in various physiological and pathological processes (Calabrese et al., 2007). As shown in Figure 4B, the three DAGs significantly promoted NO production in RAW 264.7 cells in a concentration-dependent manner. AGR and DAG-60, with similar A/G ratios, exhibited comparable abilities to stimulate NO secretion in RAW 264.7 cells. RAW 264.7 cells treated with DAG-100 secreted the highest amount of NO, reaching a maximum of 15.08 μM at a concentration of 800 μg/mL. NO production induced by DAG-140 was lower than that induced by the other samples at all concentrations tested. In summary, the ability of DAGs to stimulate NO secretion can be ranked as follows: DAG-100 (Mw = 15.0 kDa, A/G = 1:25.16) > DAG-60 (Mw = 16.7 kDa, A/G = 1:9.61) > DAG-140 (Mw = 12.9 kDa, A/G = 0). In general, the immunomodulatory effect of DAGs on RAW 264.7, is independent of Mw and A/G. Specifically, a lower A/G may confer better immunomodulatory activity to DAGs; however, complete removal of the side chain eliminates the immunomodulatory advantage of DAGs. Li et al. (2023) also showed that polysaccharide removal of side chains improved proliferation, phagocytosis, NO, and cytokine levels in Raw264.7 cells. Thus, a low A/G ratio coupled with a specific number of side chains may reflect pivotal structural characteristics that confer superior immunomodulatory activity upon DAGs. To validate this hypothesis, we evaluated the effects of the three DAGs on cytokine secretion in RAW 264.7 cells.

#### 3.5.2 Effect of AGR and DAGs on cytokine secretion by RAW 264.7 cells

Glycans with immune-enhancing effects bind to specific receptors on immune cells and stimulate cell activation, thereby promoting cytokine secretion (Baum and Cobb, 2017). TLR4 is the main receptor that stimulates macrophages and recognizes polysaccharides during polysaccharide-induced immunomodulation (Zhang et al., 2016). TLR4 activation has been shown to trigger a cascade catalytic response of signaling molecules in immune cells, which then promotes the secretion of cytokines, including TNF-α, IL-6, and others (Wang et al., 2019). These cytokines directly participate in intercellular interactions, thus achieving the effect of regulating the body’s immune response and inhibiting tumor growth. Therefore, in this study, we evaluated the immune-enhancing activity of DAGs by investigating their effects on cytokine secretion.

**FIGURE 5:**
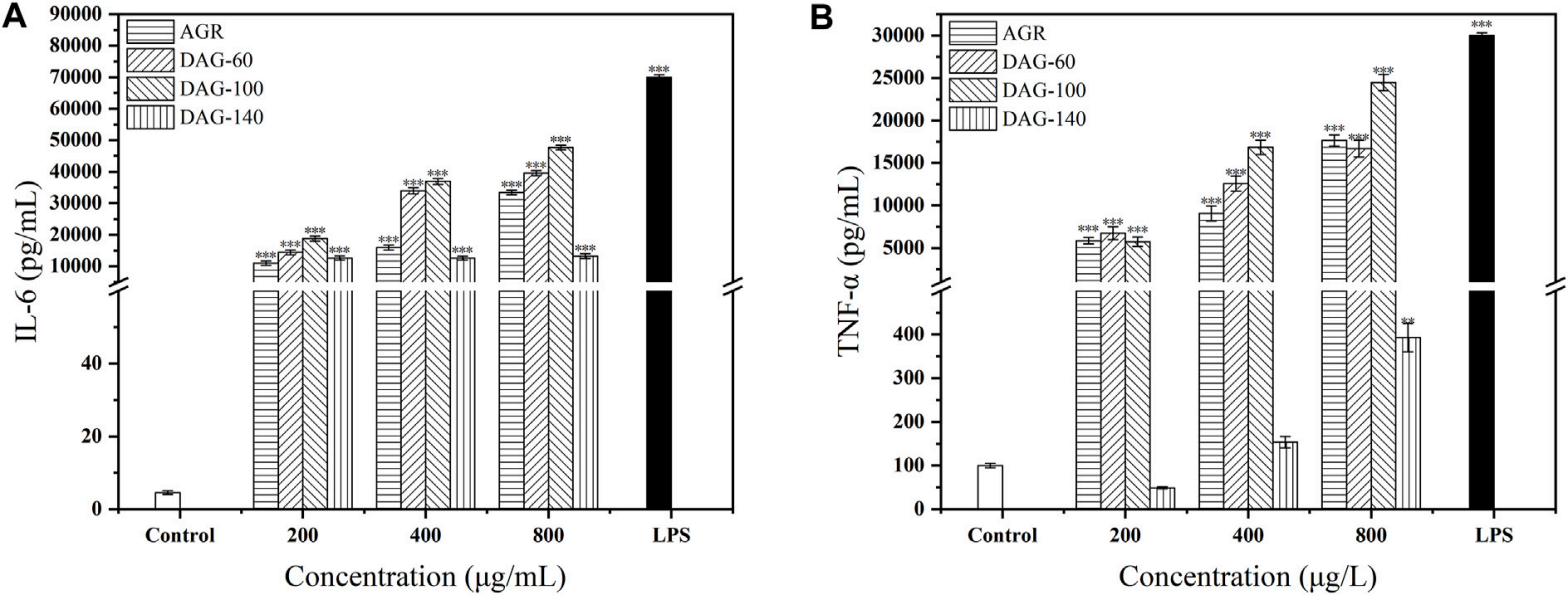
Effect of AGR and DAGs on the secretion of TNF-α **(A)** and IL-6 **(B)** in RAW 264.7 cells. *P < 0.05, **P < 0.01, ***P < 0.001 compared to the control.

AGR, DAG-60, and DAG-100 significantly increased IL-6 secretion in a concentration-dependent manner compared to the results in the control group (Figure 5A). DAG-140 had a positive effect on IL-6 secretion; however, this effect was independent of concentration. In addition, all three DAGs promoted TNF-α secretion in a concentration-dependent manner (Figure 5B). DAG-140 had a significant effect on the promotion of TNF-α secretion only at a concentration of 800 μg/mL (*P* < 0.01). The ability of DAG (at any concentration) to stimulate RAW 264.7 cytokine secretion was in the following order: DAG-100 > DAG-60 > DAG-140, which is the same order as the effect on NO secretion. Notably, DAG-140 significantly promoted IL-6 secretion at all concentrations tested (*P <* 0.001), whereas the promotion of TNF-α secretion was significant only at high concentrations (*P <* 0.01). This may have been related to the removal of the arabinose moiety from the side chains. As DAG-60 and DAG-100 with arabinose side chains significantly promote TNF-α and IL-6 secretion at all concentrations tested. The key role of arabinose as an important glycosyl unit in immune enhancement was also reported by Zhang et al. (2021). Specifically, polysaccharides derived from the rhizomes of Rhizoma Ligustici Chuanxiong, which had undergone de-arabinosylation, secreted a reduced quantity of immunoregulatory factors relative to their arabinose-retaining counterparts, demonstrating diminished immunoenhancing capabilities. This observation further confirms that low A/G and the presence of an arabinosyl portion is an important structural feature of DAGs. We found that cytokine secretion was significant after treating RAW 264.7 cells with 800 μg/mL AGR and DAGs. Therefore, we chose 800 μg/mL of polysaccharide for subsequent experiments.

### 3.6 Effect of TAK-242 on the immunomodulatory activity of AGR and DAGs

**FIGURE 6:**
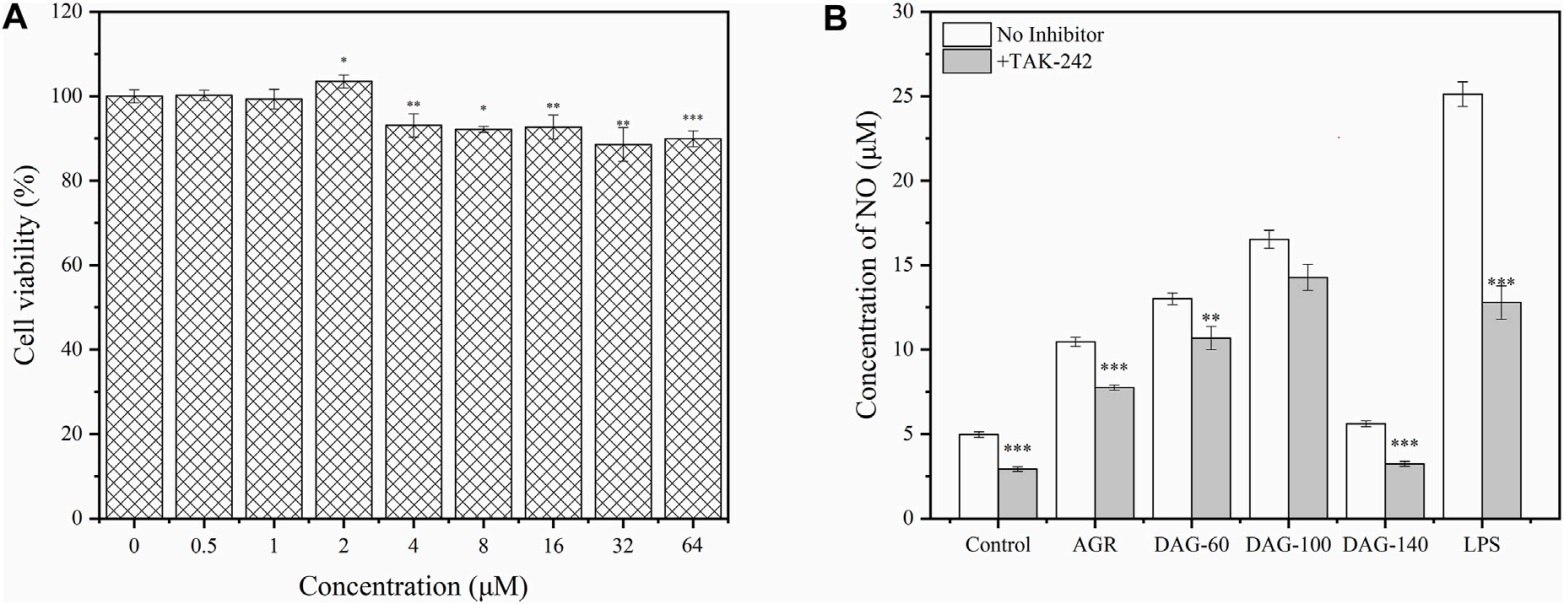
**(A)** Effect of TAK-242 with different concentrations (0–64 μM) on cell viability. **(B)** Effect of AGR and DAGs with 800 μg/mL on the production in RAW 264.7 cells incubated with or without TAK-242. *P < 0.05, **P < 0.01, ***P < 0.001 compared to the corresponding experimental group without TAK-242.

To study the resistance of DAGs to immune antagonists, we selected TAK-242, a commonly used TLR4 antagonist, to study its effect of TAK-242 on the immunomodulatory activity of the DAGs (Takashima et al., 2009). Before investigating the correlation between TLR4 and AG degradation product-mediated immunomodulatory activity, appropriate treatment concentrations of TAK-242 were selected using the CCK-8 assay. As shown in Figure 5A, cell viability gradually decreased with increasing TAK-242 concentrations. At treatment concentrations ranging from 0.5 to 2 μM, TAK-242 did not exhibit significant cytotoxicity against RAW 264.7 cells. Based on this observation, we chose 2 μM of TAK-242 for subsequent experiments. The promotion of NO secretion by AG, DAG-60, and DAG-140 was significantly inhibited by TAK-242 (*P* < 0.01). Only DAG-100-mediated NO secretion was not affected by TAK-242 pretreatment (Figure 6). This finding may be attributed to the ability of arabinose to interact with other pathways on the membrane surface of RAW 264.7 cells, thereby stimulating NO secretion. Zhao et al. (2022) noted that TLR4 recognizes hexose more so than pentose; the receptor on the membrane surface recognizes pentose, activating the NO secretion pathway. However, AGR and DAG-60, which also contain arabinoxylans, were affected by TAK-242. This could be attributed to the high A/G of AGR and DAG-60, which may be unfavorable for receptor recognition. This further confirmed the advantage of a low A/G in the structure of DAGs.

**FIGURE 7:**
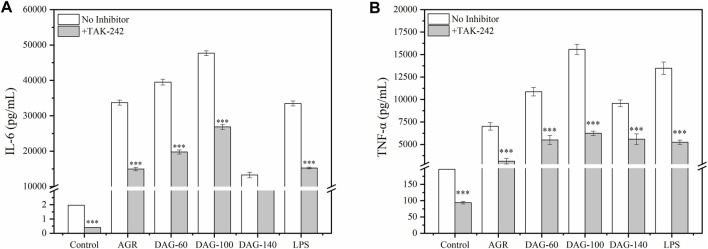
Effect of AGR and DAGs. The secretion of TNF-α **(A)** and IL-6 **(B)** in RAW 264.7 cells cultivated with or without TAK-242. n = 3, significance was determined via ANOVA. *P < 0.05, **P < 0.01, ***P < 0.001 compared to the control.

However, when TAK-242 was added, the promotion of TNF-α and IL-6 secretion by both AGR and DAGs was significantly inhibited (*P* > 0.001) and the low A/G advantage of DAG-100 was lost (Figure 7). This suggests that a low A/G may also limit the recognition of arabinose by the membrane receptor of RAW 264.7 cells, indicating that the optimal A/G value needs to be further explored. In this study, the inhibition of cytokine secretion also suggests that AGR as well as DAGs play an immune-enhancing role by activating the TLR4 pathway. Interestingly, cytokines, namely IL-6 and TNF-α,were still secreted when TLR4 was inhibited. The results of a previous study have shown that galactose, a hexose sugar, exhibits a tendency to activate TLR4, thus enhancing immune responses; however, arabinose, a pentose sugar, is inclined to engage with other receptors to achieve immunopotentiating effect (Wang et al., 2021). An active polysaccharide isolated from longan pulp, with main linkages of (1→4)-β-Glc and (1→6)-β-Man in its sugar residues, was shown to partially induce macrophage activation via the TLR2- and TLR4-mediated MyD88/IRAK4-TRAF6 signaling pathways (Rong et al., 2019). Similarly, Yuan et al., extracted a polysaccharide rich in arabinose and galactose residues from Sambucus adnata, demonstrating its capability to activate TLR2 receptors on immune cell surfaces and thus exert immunomodulatory effects (Yuan et al., 2022). Based on these findings, it is speculated that besides TLR4, other receptors such as TLR2 on the cell surface of RAW 264.7 cells may also participate in the immune enhancement process mediated by AGRs and DAGs.

Overall, we evaluated the immune-enhancing effect of AGR and DAGs on RAW 264.7 cells and found that DAG-100, which retained a side chain and had a low A/G value, exhibited the strongest immune-enhancing effect. DAG-100 was still able to promote NO secretion in RAW 264.7 cells in the presence of an immune antagonist, but the promotion of cytokine secretion was significantly inhibited. Therefore, further studies are required to optimize the A/G value.

## 4 Conclusion

In this study we applied the combined H_2_O_2_-V_C_ degradation of AGR to prepare three degraded polysaccharides, DAG-60, DAG-100, and DAG-140, at different degradation temperatures. The monosaccharide species of the three degraded polysaccharides were consistent with those of the undegraded polysaccharides, but there were differences in the arabinose-to-galactose ratio (A/G). The structure of the main functional groups of the polysaccharides did not change after H_2_O_2_-V_C_ degradation. The arabinose on the branched chain was removed completely with an increase in the degradation temperature and was almost completely removed during the degradation at 140°C. The immunity activity of AGR and the DAGs was evaluated in macrophages using the RAW 264.7 *in vitro* model. It was found that DAG-100, which retained a side chain and had a low A/G, exhibited the strongest immune-enhancing effect. DAG-100 was able to promote NO secretion in RAW 264.7 cells in the presence of an immune antagonist. Thus, having a lower A/G in the presence of side chains is the optimal structure for AGR degradation products.

## Data Availability

The original contributions presented in the study are included in the article/Supplementary Material, further inquiries can be directed to the corresponding author.

## References

[B1] Ai L. Wu J. Che N. Wu Y. Cui S. W. (2012). Extraction, partial characterization and bioactivity of polysaccharides from boat-fruited sterculia seeds. Int. J. Biol. Macromol. 51, 815–818. 10.1016/j.ijbiomac.2012.08.006 22910576

[B2] Bai X. Sadia S. Jinghua Y. (2021). Community composition and structure along environmental gradients of larix gmelinii forest in northeast China. Pak. J. Bot. 53. 10.30848/pjb2021-5(24)

[B3] Baum L. G. Cobb B. A. (2017). The direct and indirect effects of glycans on immune function. Glycobiology 27 (7), 619–624. 10.1093/glycob/cwx036 28460052

[B4] Calabrese V. Mancuso C. Calvani M. Rizzarelli E. Butterfield D. A. Giuffrida Stella A. M. (2007). Nitric oxide in the central nervous system: neuroprotection versus neurotoxicity. Nat. Rev. Neurosci. 8 (10), 766–775. 10.1038/nrn2214 17882254

[B5] Chen F. Huang G. (2018). Preparation and immunological activity of polysaccharides and their derivatives. Int. J. Biol. Macromol. 112, 211–216. 10.1016/j.ijbiomac.2018.01.169 29382579

[B6] Chen S. Liu H. Yang X. Li L. Qi B. Hu X. (2020). Degradation of sulphated polysaccharides from grateloupia livida and antioxidant activity of the degraded components. Int. J. Biol. Macromol. 156, 660–668. 10.1016/j.ijbiomac.2020.04.108 32320801

[B7] Chen X. Sun-Waterhouse D. Yao W. Li X. Zhao M. You L. (2021). Free radical-mediated degradation of polysaccharides: mechanism of free radical formation and degradation, influence factors and product properties. Food Chem. 365, 130524. 10.1016/j.foodchem.2021.130524 34252626

[B8] Chen Y. Li X.-H. Zhou L.-Y. Li W. Liu L. Wang D.-D. (2017). Structural elucidation of three antioxidative polysaccharides from tricholoma lobayense. Carbohydr. Polym. 157, 484–492. 10.1016/j.carbpol.2016.10.011 27987953

[B9] Cheng J. Huang C. Zhan Y. Liu X. Wang J. Huang C. (2023). A novel mineral-acid free biphasic deep eutectic solvent/γ-valerolactone system for furfural production and boosting the enzymatic hydrolysis of lignocellulosic biomass. Bioresour. Technol. 387, 129653. 10.1016/j.biortech.2023.129653 37573979

[B10] Cheng J. Wei C. Li W. Wang Y. Wang S. Huang Q. (2021). Structural characteristics and enhanced biological activities of partially degraded arabinogalactan from larch sawdust. Int. J. Biol. Macromol. 171, 550–559. 10.1016/j.ijbiomac.2021.01.039 33444654

[B11] Cicinskas E. Begun M. A. Tiasto V. A. Belousov A. S. Vikhareva V. V. Mikhailova V. A. (2019). *In vitro* antitumor and immunotropic activity of carrageenans from red algae chondrus armatus and their low‐molecular weight degradation products. J. Biomed. Mater. Res. Part A 108 (2), 254–266. 10.1002/jbm.a.36812 31606930

[B12] Consumi M. Tamasi G. Pepi S. Leone G. Bonechi C. Magnani A. (2022). Analytical composition of flours through thermogravimetric and rheological combined methods. Thermochim. Acta 711, 179204. 10.1016/j.tca.2022.179204

[B13] Curcio M. Puoci F. Iemma F. Parisi O. I. Cirillo G. Spizzirri U. G. (2009). Covalent insertion of antioxidant molecules on chitosan by a free radical grafting procedure. J. Agric. Food Chem. 57 (13), 5933–5938. 10.1021/jf900778u 19566085

[B14] Currier N. L. Lejtenyi D. Miller S. C. (2003). Effect over time of *in-vivo* administration of the polysaccharide arabinogalactan on immune and hemopoietic cell lineages in murine spleen and bone marrow. Phytomedicine 10 (2-3), 145–153. 10.1078/094471103321659852 12725568

[B15] Drouillard S. Poulet L. Marechal E. Amato A. Buon L. Loiodice M. (2022). Structure and enzymatic degradation of the polysaccharide secreted by nostoc commune. Carbohydr. Res. 515, 108544. 10.1016/j.carres.2022.108544 35367699

[B16] Fan Z. Xu S. Huang C. Cao Y. Wu X. (2023). Dual preservative strategy for facilitating bamboo durability using cinnamaldehyde and diethylenetriamine and its reaction characteristics on bamboo cell wall. Industrial Crops Prod. 206, 117600. 10.1016/j.indcrop.2023.117600

[B17] Gao X. Kumar R. Wyman C. E. (2014). Fast hemicellulose quantification via a simple one‐step acid hydrolysis. Biotechnol. Bioeng. 111 (6), 1088–1096. 10.1002/bit.25174 24343864

[B18] Gong G. Dang T. Deng Y. Han J. Zou Z. Jing S. (2018). Physicochemical properties and biological activities of polysaccharides from lycium barbarum prepared by fractional precipitation. Int. J. Biol. Macromol. 109, 611–618. 10.1016/j.ijbiomac.2017.12.017 29222018

[B19] Guo W. Rao G. H. Wen X. (2021). Arabinogalactan in banana: chemical characterization and pharmaceutical effects. Int. J. Biol. Macromol. 167, 1059–1065. 10.1016/j.ijbiomac.2020.11.060 33188809

[B20] Hu B. B. Zhang S. L. Wang Z. Y. Han Q. Y. Zhang D. S. Zheng Y. G. (2023). Degradation method, structural characteristics, biological activity and structure-activity relationship of degraded polysaccharides. Food Rev. Int., 1–30. 10.1080/87559129.2023.2273933

[B21] Li J. Li S. Zheng Y. Zhang H. Chen J. Yan L. (2019). Fast preparation of rhamnogalacturonan I enriched low molecular weight pectic polysaccharide by ultrasonically accelerated metal-free fenton reaction. Food Hydrocoll. 95, 551–561. 10.1016/j.foodhyd.2018.05.025

[B22] Li M. Ma F. Li R. Ren G. Yan D. Zhang H. (2020). Degradation of tremella fuciformis polysaccharide by a combined ultrasound and hydrogen peroxide treatment: process parameters, structural characteristics, and antioxidant activities. Int. J. Biol. Macromol. 160, 979–990. 10.1016/j.ijbiomac.2020.05.216 32473217

[B23] Li Z. Wang M. Yang Z. (2023). Structural characterization, anti-tumor and immunomodulatory activity of intracellular polysaccharide from armillaria luteo-virens. Carbohydr. Res. 534, 108945. 10.1016/j.carres.2023.108945 37738818

[B24] Liang M. Deng J. Gu J. Yang J. Ge F. Huang C. (2023). TMBPF-induced neurotoxicity and oxidative stress in zebrafish larvae: impacts on central nervous system development and dopamine neurons. Ecotoxicol. Environ. Saf. 268, 115710. 10.1016/j.ecoenv.2023.115710 38000302

[B25] Liang S. Liao W. Ma X. Li X. Wang Y. (2017). H_2_O_2_ oxidative preparation, characterization and antiradical activity of a novel oligosaccharide derived from flaxseed gum. Food Chem. 230, 135–144. 10.1016/j.foodchem.2017.03.029 28407893

[B26] Lo T. C.-T. Chang C. A. Chiu K.-H. Tsay P.-K. Jen J.-F. (2011). Correlation evaluation of antioxidant properties on the monosaccharide components and glycosyl linkages of polysaccharide with different measuring methods. Carbohydr. Polym. 86 (1), 320–327. 10.1016/j.carbpol.2011.04.056

[B27] Ma C. Bai J. Shao C. Liu J. Zhang Y. Li X. (2021). Degradation of blue honeysuckle polysaccharides, structural characteristics and antiglycation and hypoglycemic activities of degraded products. Food Res. Int. 143, 110281. 10.1016/j.foodres.2021.110281 33992381

[B28] Machado F. Gómez-Domínguez I. Hurtado-Ribeira R. Martin D. Coimbra M. del Castillo M. (2024). *In vitro* human colonic fermentation of coffee arabinogalactan and melanoidin-rich fractions. Int. J. Biol. Macromol. 275, 133740. 10.1016/j.ijbiomac.2024.133740 38986986

[B29] Ofoedu C. E. You L. J. Osuji C. M. Iwouno J. O. Kabuo N. O. Ojukwu M. (2021). Hydrogen peroxide effects on natural-sourced polysacchrides: free radical formation/production, degradation process, and reaction mechanism-a critical synopsis. Foods 10 (4), 699. 10.3390/foods10040699 33806060 PMC8064442

[B30] Rakhmanberdyeva R. K. Zhauynbayeva K. S. Senchenkova S. N. Shashkov A. S. Bobakulov K. M. (2019). Structure of arabinogalactan and pectin from the silybum marianum. Carbohydr. Res. 485, 107797. 10.1016/j.carres.2019.107797 31494303

[B31] Rong Y. Yang R. L. Yang Y. Z. Wen Y. Z. Liu S. X. Li C. F. (2019). Structural characterization of an active polysaccharide of longan and evaluation of immunological activity. Carbohydr. Polym. 213, 247–256. 10.1016/j.carbpol.2019.03.007 30879666

[B32] Sun Y. Yang B. Wu Y. Liu Y. Gu X. Zhang H. (2015). Structural characterization and antioxidant activities of kappa-carrageenan oligosaccharides degraded by different methods. Food Chem. 178, 311–318. 10.1016/j.foodchem.2015.01.105 25704717

[B33] Takashima K. Matsunaga N. Yoshimatsu M. Hazeki K. Kaisho T. Uekata M. (2009). Analysis of binding site for the novel small-molecule TLR4 signal transduction inhibitor TAK-242 and its therapeutic effect on mouse sepsis model. Br. J. Pharmacol. 157 (7), 1250–1262. 10.1111/j.1476-5381.2009.00297.x 19563534 PMC2743844

[B34] Tang S. Jiang M. Huang C. Lai C. Fan Y. Yong Q. (2018). Characterization of arabinogalactans from larix principis-rupprechtii and their effects on NO production by macrophages. Carbohydr. Polym. 200, 408–415. 10.1016/j.carbpol.2018.08.027 30177181

[B35] Trigui I. Yaich H. Sila A. Cheikh-Rouhou S. Bougatef A. Blecker C. (2018). Physicochemical properties of water-soluble polysaccharides from black cumin seeds. Int. J. Biol. Macromol. 117, 937–946. 10.1016/j.ijbiomac.2018.05.202 29864536

[B36] Wang N. Liang H. Zen K. (2014). Molecular mechanisms that influence the macrophage m1-m2 polarization balance. Front. Immunol. 5, 614. 10.3389/fimmu.2014.00614 25506346 PMC4246889

[B37] Wang W. Deng Z. Wu H. Zhao Q. Li T. Zhu W. (2019). A small secreted protein triggers a TLR2/4-dependent inflammatory response during invasive candida albicans infection. Nat. Commun. 10 (1), 1015. 10.1038/s41467-019-08950-3 30833559 PMC6399272

[B38] Wang X. Huang C. Fu X. Jeon Y. J. Mao X. Wang L. (2023). Bioactivities of the popular edible brown seaweed sargassum fusiforme: a review. J. Agric. Food Chem. 71 (44), 16452–16468. 10.1021/acs.jafc.3c05135 37876153

[B39] Wang X. Huang C. Yang F. Wang K. Cha S.-H. Mao X. (2024). Fucoidan isolated from the edible seaweed sargassum fusiforme suppresses skin damage stimulated by airborne particulate matter. Algal Res. 77, 103339. 10.1016/j.algal.2023.103339

[B40] Wang Y. Sun J. Xue L. Liu J. Nie C. Fan M. (2021). l-Arabinose attenuates gliadin-induced food allergy via regulation of Th1/Th2 balance and upregulation of regulatory T cells in mice. J. Agric. Food Chem. 69 (12), 3638–3646. 10.1021/acs.jafc.0c07167 33734700

[B41] Xu Y. Niu X. Liu N. Gao Y. Wang L. Xu G. (2018). Characterization, antioxidant and hypoglycemic activities of degraded polysaccharides from blackcurrant (Ribes nigrum L.) fruits. Food Chem. 243, 26–35. 10.1016/j.foodchem.2017.09.107 29146337

[B42] Yao X. C. Cao Y. Pan S. K. Wu S. J. (2013). Preparation of peach gum polysaccharides using hydrogen peroxide. Carbohydr. Polym. 94 (1), 88–90. 10.1016/j.carbpol.2013.01.048 23544514

[B43] Yuan D. Li C. Huang Q. Fu X. (2020). Ultrasonic degradation effects on the physicochemical, rheological and antioxidant properties of polysaccharide from sargassum pallidum. Carbohydr. Polym. 239, 116230. 10.1016/j.carbpol.2020.116230 32414439

[B44] Yuan L. Zhong Z. C. Liu Y. Quan H. Lu Y. Z. Zhang E. H. (2022). Structures and immunomodulatory activity of one galactose- and arabinose-rich polysaccharide from sambucus adnata. Int. J. Biol. Macromol. 207, 730–740. 10.1016/j.ijbiomac.2022.03.132 35346678

[B45] Zhang S. An L. Li Z. Wang X. Wang H. Shi L. (2021). Structural elucidation of an immunological arabinan from the rhizomes of ligusticum chuanxiong, a traditional chinese medicine. Int. J. Biol. Macromol. 170, 42–52. 10.1016/j.ijbiomac.2020.12.069 33316344

[B46] Zhang Z. S. Wang X. M. Zhao M. X. Qi H. M. (2014). Free-radical degradation by Fe^2+^/Vc/H_2_O_2_ and antioxidant activity of polysaccharide from tremella fuciformis. Carbohydr. Polym. 112, 578–582. 10.1016/j.carbpol.2014.06.030 25129784

[B47] Zhang X. Qi C. Guo Y. Zhou W. Zhang Y. (2016). Toll-like receptor 4-related immunostimulatory polysaccharides: primary structure, activity relationships, and possible interaction models. Carbohydr. Polym. 149, 186–206. 10.1016/j.carbpol.2016.04.097 27261743

[B48] Zhang X. R. Qi C. H. Cheng J. P. Liu G. Huang L. J. Wang Z. F. (2014). Lycium barbarum polysaccharide LBPF4-OL may be a new Toll-like receptor 4/MD2-MAPK signaling pathway activator and inducer. Int. Immunopharmacol. 19 (1), 132–141. 10.1016/j.intimp.2014.01.010 24462389

[B49] Zhao M. Hou J. Zheng S. Ma X. Fu X. Hu S. (2022). Peucedanum praeruptorum dunn polysaccharides regulate macrophage inflammatory response through TLR2/TLR4-mediated MAPK and NF-κB pathways. Biomed. and Pharmacother. 152, 113258. 10.1016/j.biopha.2022.113258 35709651

[B50] Zheng L. Ma Y. Zhang Y. Meng Q. Yang J. Wang B. (2021). Increased antioxidant activity and improved structural characterization of sulfuric acid-treated stepwise degraded polysaccharides from pholiota nameko PN-01. Int. J. Biol. Macromol. 166, 1220–1229. 10.1016/j.ijbiomac.2020.11.004 33157137

[B51] Zou M. Y. Nie S. P. Yin J. Y. Xie M. Y. (2020). Ascorbic acid induced degradation of polysaccharide from natural products: a review. Int. J. Biol. Macromol. 151, 483–491. 10.1016/j.ijbiomac.2020.02.193 32084460

