# Supplementary material for: Effect of H2O2-VC degradation on structural characteristics and immunomodulatory activity of larch arabinogalactan
Source: Front Bioeng Biotechnol. 2024 Aug 7;12:1461343. doi: 10.3389/fbioe.2024.1461343 (PMC11335654; doi:10.3389/fbioe.2024.1461343)
Supplement: Supplementary file 1 [file DataSheet1.docx]

Effect of H_2_O_2_-V_C_ degradation on structural characteristics and immunomodulatory activity of larch arabinogalactan

Huimin Qi^a^, Shuo Tang^b^, Bin Bian^a^, Chenhuan Lai^a^, Yanan Chen^a^, Zhe Ling^a^, Qiang Yong^a *^

^a^Jiangsu Co-Innovation Center of Efficient Processing and Utilisation of Forest Resources, College of Chemical Engineering, Nanjing Forestry University, Nanjing 210037, People’s Republic of China

^b^Nanjing Institute of Comprehensive Utilization of Wild Plants, Nanjing 211111, People’s Republic of China





Figure S1 The content and degradation ratio of monosaccharides in H_2_O_2_ concentration gradient

Degradation Ratio : Mass difference between pre- and post-degradation mass/pre-degradation mass





Figure S2 The content and degradation ratio of monosaccharides in Vc concentration gradient





Figure S3 The content and degradation ratio of monosaccharides in time gradient





Figure S4 The content and degradation ratio of monosaccharides in temperature gradient





Figure S5 Maximum absorption wavelength of polysaccharide-Congo red complex at different concentrations of NaOH
